# Recollection and familiarity support auditory working memory in a manner analogous to visual working memory

**DOI:** 10.1016/j.cognition.2024.105987

**Published:** 2024-10-26

**Authors:** Chris Hawkins, Jon Venezia, Edward Jenkins, Sharon Li, Andrew Yonelinas

**Affiliations:** a Department of Psychology, University of California, Davis, 1 Shields Avenue, Davis, CA 95616, USA; b Department of Otolaryngology & Head/Neck Surgery, Loma Linda University, Loma Linda, CA 92350, USA

**Keywords:** Working memory, Recollection, Familiarity, Auditory cognition, Auditory working memory, Dual-process

## Abstract

Prior work has suggested that visual working memory as measured in change detection tasks can be based on *recollection*, whereby participants consciously identify a specific feature of a stimulus that has changed, or on *familiarity*, whereby participants sense that a change has occurred but are unable to consciously access what has changed. Whether recollection and familiarity also contribute to auditory working memory is unclear. The present study aims to address that gap in knowledge by having participants make confidence judgments in change detection tests for speech sounds and pure tones. The results indicated that both recollection and familiarity contribute to auditory working memory across a variety of conditions, and showed that these two processes are functionally dissociable. With speech sounds, subjects were better able to detect syllable changes compared to tone or location changes, and this benefit reflected a selective increase in recollection rather than familiarity. Moreover, for pure tones, both recollection and familiarity also contributed to performance, but recollection was found to be selectively eliminated under stimulus-limited test conditions (i.e., noise-masked, brief dichotic presentations). The results indicate that recollection and familiarity contribute to auditory working memory in a manner that is functionally similar to that observed in visual working memory.

## 1. Introduction

Working memory reflects the ability to temporarily maintain and process relevant information from our environment. This ability has been the focus of extensive study because it is critical for many important cognitive tasks such as scene perception, spoken language processing, and reading (Baddeley, 2006; Cowan, 2017; Miyake & Friedman, 2013; D’Esposito & Postle, 2015; Hasher & Zacks, 1988), and it is disrupted in a variety of different populations such as in healthy aging and various types of neurological patients (Anders et al., 1972; Goodrich et al., 2019; Lee & Park, 2005; Salthouse & Babcock, 1991; Warrington & Shallice, 1972). A common method for assessing working memory is the change detection task, in which a stimulus is presented then after a very brief delay the same stimulus, or a changed version of the stimulus, is presented and subjects must indicate if the two stimuli were the same or different. Recent work has indicated that visual change detection performance can be supported by two separable memory processes: *recollection*, whereby participants consciously identify a specific feature of a stimulus that has changed, and *familiarity,* whereby participants sense that a change has occurred but are unable to consciously access what has changed (e.g., Davidoff & Ostergaard, 1984; Aly & Yonelinas, 2012; Goodrich & Yonelinas, 2016; Goodrich et al., 2019; Yonelinas et al., 2024; for review, see Yonelinas, 2023). However, this work has been limited almost exclusively to visual materials, and so whether recollection and familiarity also contribute to auditory forms of working memory is currently unclear.

One possibility is that recollection and familiarity play similar roles in both auditory and visual working memory. Alternatively, the processes supporting visual and auditory working memory may be quite different. In support of the latter possibility, a large body of research has indicated that at least some forms of auditory working memory such as memory for phonological information are functionally and neuroanatomically distinct from working memory for visual information (Baddeley & Hitch, 1974; for review see Baddeley & Hitch, 1994). In addition, auditory working memory for non-phonological information, such as for tones, can be quite different from working memory for speech-based phonological information (e.g., Lad et al., 2020), and so the extent to which recollection and familiarity contribute to different forms of auditory working memory could also differ from one another.

**Fig. 1.**
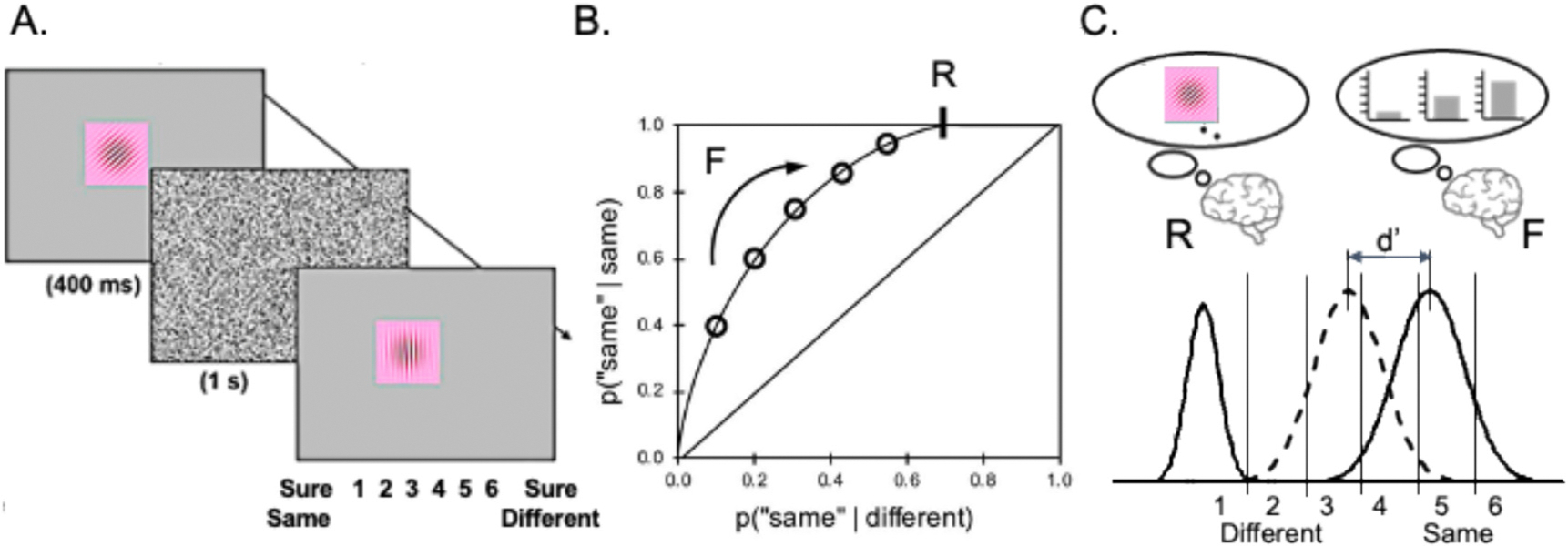
A) A visual change detection task in which subjects indicate their confidence that a test stimulus was different from or the same as a study stimulus. B) A receiver operating characteristic plotting hit rate (i.e., the proportion of same trials correctly identified as same) against false alarm rate (i.e., the proportion of different trials incorrectly identified as same) as a function of confidence. The observed ROCs are typically curvilinear indicating that the same trials are more familiar than the changed trials. In addition, the ROCs are asymmetrical such that the function appears to intersect the upper x-axis, indicating that for some proportion of changed trials the subject recollects that a feature has changed. C) A model of the underlying memory strength distributions for recollection (R) and familiarity (F; which is measured as a d’ value), and how they are mapped onto confidence.

To assess the memory processes underlying visual working memory a number of studies have examined Receiver Operating Characteristics (ROCs) (for reviews of this method see Yonelinas & Parks, 2007; Macmillan & Creelman, 2005). The general methods, results, and the associated measurement model underlying these studies are illustrated in Fig. 1 (from Goodrich et al., 2019; for reviews see Yonelinas, 2023; Yonelinas et al., 2024). For example, in a change detection task for visual gradients, subjects might be required to rate their confidence that a test stimulus was the same or different from a studied stimulus on a six-point scale (Fig. 1A). ROCs can then be plotted (Fig. 1B) such that each point represents performance (i.e., hits vs. false alarms on the y- and x-axis, respectively) at each level of confidence. In the present experiments, the left-most point reflects the proportion of items that the subject was sure was the ‘same’ as the studied item (i.e., they received a ‘1’ response) whereas each of the subsequent points include the next most confident responses (i.e., the second point reflects the cumulative proportion of 1 s and 2 s). Thus, the function shows performance as response criterion is varied, with the left-most point reflecting performance when subjects are confident that the test item was the same, and the right-most point reflecting performance when subjects are confident that the test item was different. The area under the observed ROC reflects overall discrimination such that a value of 1.0 reflects perfect performance and 0.5 reflects chance-level performance.

The observed ROCs in visual working memory are typically curvilinear downward (i.e., an inverted U shape). In addition, they are asymmetrical such that the function appears to intersect the upper x-axis rather than approach the (1,1) intercept (Fig. 1B; for review see Yonelinas, 2023). Recollection and familiarity can be estimated by fitting a dual process signal detection model (Fig. 1C; Yonelinas, 1994) to the observed ROC. The model assumes that changed items (i.e., the dashed distribution) will be less familiar than non-changed items (i.e., the solid distribution to the right), such that higher levels of familiarity will lead to higher confidence that the test stimulus was the same as the study stimulus. Moreover, if subjects can recollect that a specific feature has changed (e.g., if they notice that the angle, the frequency, or the color of the visual gradient changed) this is expected to lead to a high confidence response that the item was different (i.e., the solid distribution on the left). This will lead to an increase in the proportion of change trials that receive a high confidence ‘different’ response, which will be reflected in the upper right point on the ROC shifting away from the (1,1) intercept. This will push the ROC to the left and produce an asymmetrical ROC. In this way, the curvilinearity of the observed ROC is indicative of familiarity whereas the asymmetry is indicative of recollection.

Note that if recollection is zero, the dual process signal detection model collapses into a single process model (i.e., the equal variance signal detection model; Swets et al., 1961). In addition, a closely related model is the unequal variance signal detection model that includes the same familiarity process, but also includes a variance ratio parameter which allows the familiarity distribution of the changed items to have greater variability than the familiarity distribution of the same items. This produces ROCs that are very similar in shape to the dual process model, and it generally supports similar conclusions (for a comparison of these models see Yonelinas & Parks, 2007). The greater variance of the changed items can be thought of as reflecting two processes that influence the ‘different’ trials (e.g., recollection and familiarity) but only one influences the ‘same’ trials (e.g., familiarity). We will focus on the dual process model, but will consider the results in light of each of these alternative models in the discussion.

Prior studies of visual working memory ROCs have indicated that recollection and familiarity-based discriminations can be functionally separated. For example, the degree of ROC curvilinearity (i.e., familiarity) and the degree of ROC asymmetry (i.e., recollection) can be dissociated, indicating that performance cannot be accurately characterized by a single memory strength parameter. For example, Aly and Yonelinas (2012) found that in a change detection task for visual scenes, when the changes were global in nature such that the entire image was pinched or expanded very slightly, highly curved ROCs that were only slightly asymmetrical were observed. On the other hand, when the changes involved discrete manipulations such as the addition or removal of an object within a scene, the ROCs became less curved and exhibited a high degree of asymmetry. In addition, whereas both recollection and familiarity parameters were needed to produce the ROCs in most conditions, the contribution of recollection could be effectively eliminated under stimulus-limited conditions. That is, when the visual stimuli were briefly presented to different visual fields and were masked with visual noise, the ROCs were curved and symmetrical, rather than asymmetrical, suggesting that subjects were unable to use recollection as a basis for responding under these conditions. In addition to these functional dissociations, subsequent visual working memory studies have indicated that recollection and familiarity are related to distinct subjective states (i.e., related to a subjective feeling of ‘perceiving a specific change’ vs simply ‘sensing’ that a change occurred) and involve partially distinct brain networks (Aly, Ranganath and Yonelinas, (2013); Aly and Yonelinas (2012); Aly et al. (2011); Rensink (2004)).

Do recollection and familiarity also contribute to auditory working memory? Although there have been numerous studies examining the role of recollection and familiarity in visual working memory, we are aware of only one previous ROC study examining recollection and familiarity in auditory working memory. In a study by McAnally et al. (2010), subjects were presented with complex auditory scenes containing up to 8 environmental sounds (e.g., siren, flute, voice, etc.). After a brief delay, the same auditory scene or a scene with one sound removed was presented, and subjects made a 6-point confidence response indicating if the two auditory scenes were the same or different. The resulting ROCs were curved and asymmetrical, which is consistent with the notion that both recollection and familiarity contributed to performance. However, that study leaves open three important questions that the current study was designed to address.

First, do recollection and familiarity reflect functionally distinct processes in auditory working memory? If so, it should be possible to find experimental manipulations that influence one of the processes without influencing the other. Alternatively, they may simply reflect two aspects of the same underlying memory signal that always increase or decrease together. For example, recollection and familiarity may reflect strong and weak memories, respectively, such that any manipulation that increases overall performance should increase both recollection and familiarity. Second, are the recollection and familiarity processes that support auditory working memory functionally similar to those that are found to support visual working memory? In visual working memory, presenting stimuli under more challenging ‘stimulus-limited’ conditions (i.e., visually masked and rapidly presented to different visual fields), results in a selective reduction in recollection (Aly & Yonelinas, 2012). Whether a similar dissociation is observed in auditory working memory is currently unknown. Third, in addition to complex auditory scenes, do recollection and familiarity also contribute to other auditory stimuli like speech-based phonological materials and simple tones? In visual working memory, recollection and familiarity appear to play a very general role across various types of stimuli ranging from complex visual scenes to simpler materials like gabor gradients (Aly & Yonelinas, 2012; Goodrich et al., 2019; Goodrich & Yonelinas, 2016). Assessing whether similar effects are observed across various auditory materials is critical in determining the extent to which recollection and familiarity play a general role in auditory working memory or whether their contributions may be restricted to certain classes of auditory stimuli.

In Experiment 1, we examined change detection for speech sounds in which we either changed the vowel sounds (i.e., ‘beet’ vs ‘bait’; hereafter referred to as a “syllable” change), the pitch, the perceived location, or all three features of the speech sounds. Our aim was to determine whether there is evidence of both recollection and familiarity in speech-based auditory working memory across a range of different types of auditory changes, and determine whether recollection and familiarity were dissociable, or whether they changed in similar ways across these different kinds of changes. In addition, we measured change-identification accuracy (i.e., the ability to identify which type of auditory change had occurred) in order to determine how recollection and familiarity were related to the ability to identify the specific auditory feature that had changed. Presumably, subjects who are good at detecting that a change has occurred should also be good at reporting the precise changed feature. However, how differences in recollection and familiarity relate to the change-identification accuracy in auditory working memory is not known. In Experiment 2 we examined change detection for pure tones in which we varied the pitch in order to determine whether results with speech stimuli would generalize to very simple auditory stimuli. Experiment 3 was identical to Experiment 2 except that we tested memory for tones under stimulus-limited conditions (i.e., brief dichotic presentations in the presence of a noise mask) in order to determine if these conditions would eliminate the contribution of recollection, as observed with working memory for simple visual materials (Aly & Yonelinas, 2012).

## 2. Experiment 1: working memory for speech sounds

### 2.1. Methods

#### 2.1.1. Participants

24 participants were recruited from the undergraduate population at the University of California, Davis and participated in the experiment for course credit. The study was approved by the University of California, Davis Institutional Review Board and informed consent was obtained from all participants prior to testing. All participants reported normal hearing, and informed consent was obtained prior to the study. Three subjects were removed for failing to use a sufficient range of confidence responses to evaluate ROC shape (i.e., an ROC with fewer than 3 points), resulting in a final sample of 21 participants (16 female).

#### 2.1.2. Materials

Natural utterances of the words “beet” and “bait” were recorded on an AudioTechnica AT2020 microphone, preamplified with a DBX 286 s effects processor, and digitized (44.1 kHz) with a Scarlett 2i2 USB audio interface. Recordings were made by a single male talker in a sound-attenuated chamber. The vowel portions of the recorded waveforms (diphthongs /iː/ and /eɪ/) were manually excised at zero crossings nearest the onset and offset of the periodic portion of the speech signal using Audacity and saved as separate wav files. The consonant bursts preceding and following /iː/ in the “beet” recording (/b/ and /t/, respectively) were similarly excised at zero crossings and saved as separate wav files. A 20-step continuum from /iː/ to /eɪ/ was then generated from the exemplar recordings using the STRAIGHT toolbox in MATLAB (Kawahara et al., 2001). The resultant synthesized vowel sounds were cropped at zero crossings nearest the onset and offset of speech energy, normalized to equal root-mean-square amplitude, and appended between the original recordings of /b/ and /t/ to produce a 20-step continuum from “beet” to “bait.” A custom Python script was generated to produce pitch-shifted and/or lateralized versions of the speech stimuli. Pitch shifting was accomplished using Praat via the *parselmouth* Python package (Boersma & Van Heuven, 2001). Based on examination of the natural pitch contours of the original “beet” and “bait” recordings, a closely matching stylized pitch contour was generated with F0 beginning at 135 Hz, remaining flat at 135 Hz for 60 ms, and decreasing linearly to 80 Hz over 70 ms. Shifts in the mean F0 were introduced by a simple additive offset (X Hz) applied to the entire stylized pitch contour. The stylized pitch contour was imposed on the original speech recording using the overlap-add resynthesis procedure. Lateralization was achieved by introducing an interaural time difference (X μs) as a frequency-dependent phase shift in the Fourier domain.

The stimuli consisted of 400 ms speech sounds that varied in syllable type (e.g., ‘beet/bait’), pitch (e.g., 100/111 Hz), and apparent location (e.g., shifting left/right presentation by ±100 μs). Sounds were presented through AmazonBasics on-ear headphones (model HP01-V2), with the volume fixed at −22.3 dB.

#### 2.1.3. Design

The experiment examined change detection performance for speech sounds such that subjects had to detect changes either in Syllable (i.e., ‘beet/bait’), Pitch (i.e., high/low), Location (i.e., left/right), or Multiple features (i.e., subtle changes in all three features). We chose lexical stimuli (monosyllabic words) to encourage processing of speech qua speech, whereas sublexical discrimination tasks are more likely to recruit sensorimotor and production related brain regions that are not required for auditory comprehension (Rogalsky et al., 2022). Along similar lines, we manipulated vowel sounds (diphthongs) because perception of vowel continua tends to be relatively continuous, with a high degree of sensitivity to within-category acoustical structure, whereas perception of consonant continua is more discrete leading to categorical-like “jumps” in perception that are highly task-dependent, and at least partially reliant on the speech motor system (Gerrits & Schouten, 2004; Kronrod et al., 2016; Möttönen & Watkins, 2009). Pitch and lateralization were manipulated concurrently with vowel structure because these manipulations are (i) unidimensional (i.e., linked to single acoustic cue, mean F0 and interaural time difference, respectively) and (ii) orthogonal to the manipulation of vowel structure – that is, both F0 and interaural time difference reflect the temporal and/or periodic structure of the speech signal, whereas vowel identity reflects its spectral structure. These stimuli were selected because we felt they were representative of common auditory features in speech, and because they are commonly used in audiological research (Mattingly et al., 1971; Zatorre et al., 1992; Altmann & Kamide, 2007; for review see Fastl & Zwicker, 2006). We had no theoretically driven predictions regarding whether the relative contributions of R and F would differ for the different change types, although we speculated that syllable changes may preferentially support recollection given that the syllable difference seemed more categorical in nature and thus may be more amenable to conscious reports. In addition, testing occurred either in ‘Mixed’ lists in which different types of changes were mixed randomly within a list such that subjects did not know which feature would change from trial to trial, or they were tested in ‘Blocked’ lists in which only one feature would change from trial to trial. The ‘mixed’ and ‘blocked list conditions were included to investigate whether performance would depend on whether subjects had to attend to a single feature or multiple different stimulus features, as was done in a previous study of visual working memory (Goodrich et al., 2019). However, in the current study (and in the earlier visual study) the types of lists led to similar results and so we collapsed across this factor, as explained below.

#### 2.1.4. Procedure

**Fig. 2.**
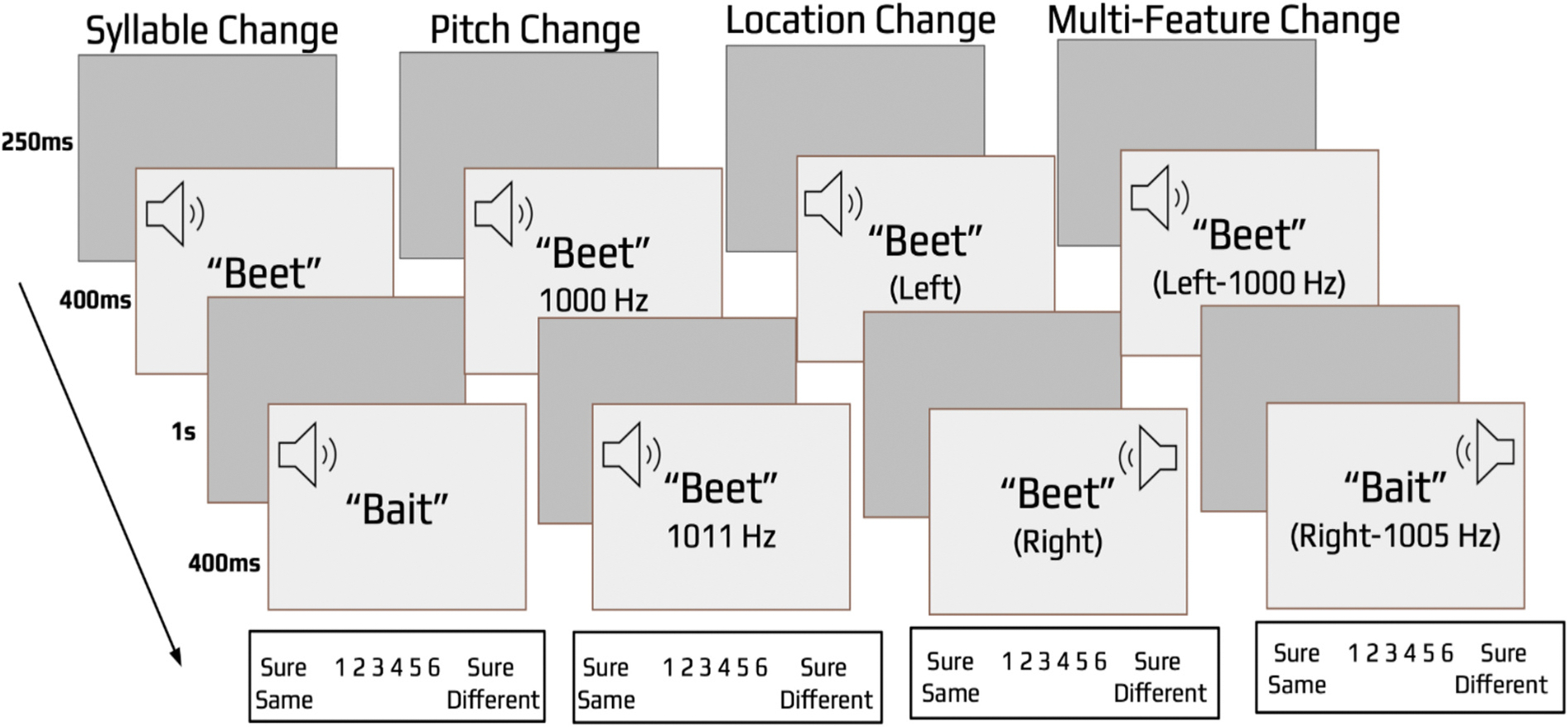
Illustration of the test procedures in Experiment 1. Subjects heard a speech sound followed by the same sound or a changed version of the sound, and they then indicated if the two sounds were the same or different using a 6-point confidence scale. Speech sounds could change in syllable, pitch, location, or a combination of all three features. Performance was examined in blocked list conditions in which only one feature would change, and in a mixed list condition which contained a randomized sequence of each change type. In the mixed list condition only, immediately following each change detection confidence response, a three-alternative, forced-choice identification judgment (not pictured) was required in which subjects indicated if there was a change in either syllable, pitch, or location.

Each trial began with a 250 ms visual fixation cross followed by a speech sound that lasted 400 ms (Fig. 2). After a 1000 ms delay, a second speech sound was presented for 400 ms. A visual confidence scale was then presented prompting subjects to make a self-paced same/different judgment using a 1 to 6 confidence scale. Each numerical option was labeled on the screen (1 = sure same, 2 = probably same, 3 = maybe same, 4 = maybe different, 5 = probably different, 6 = sure different). The scale was explained to participants prior to the experiment and each participant was familiarized with the possible change types through 15 pre-experimental practice trials that required participants to detect example changes (and same trials) for each dimension in isolation, mimicking the Blocked-trials condition; and 9 trials that required participants to detect changes among same trials in a mixed list, mimicking the Mixed-trials condition.

The experiment consisted of three sets of Blocked trials in which only one feature changed from trial to trial (i.e., Syllable, Pitch, or Location), and one set of Mixed trials in which all three features could change from trial to trial, totaling 450 trials. There were a total of 180 trials in the blocked condition. Each set of Blocked trials contained 30 ‘no change’ trials and 30 ‘change’ trials, presented in a random order. In the Syllable condition, participants heard a sound along a twenty-step continuum varying from ‘Beet’ (step 1) to ‘Bait’ (step 20). During a ‘no change’ trial, the second sound was identical to the first sound whereas for a ‘change’ trial the second sound was 8 steps removed from the first sound. In the Pitch condition, for ‘no change’ trials, both stimuli were presented at the same frequency whereas for the ‘change’ trials, the second sound stimulus was always presented with an increase of 11 Hz from its original pitch value (e.g., 100 Hz to 111 Hz), on a continuum from −30 (i.e., 105 Hz) to +19 (i.e., 124 Hz), so that the maximum possible pitch value was 135 Hz. Lastly, in the Location condition, for a ‘no change’ trial, the sounds were presented on the same side (e.g., left/left) whereas for a ‘change’ trial and the location of the second item was shifted from left to right or from right to left (i.e., the timing of sound presentation shifted by ±100 μs (e.g., from −70 ms (left lateralized) to +30 ms (right lateralized) on a continuum bound between −100 ms and + 100 ms). For ‘change’ and ‘no change’ trials, sound presentation was not always centered; rather, sounds were presented at equal points on the above-described continuums for all dimensions within a trial, varying from sound 1 to sound 2 only on ‘change’ trials and only in the relevant dimension (i.e., syllable, pitch, or location). The Mixed block contained a random mixture of syllable, pitch, location, and multiple-feature change trials. There were a total of 270 trials in the mixed block: 90 were ‘no change’ trials, 30 were syllable change trials, 30 were pitch change trials, 30 were location change trials, and 90 were multiple-feature change trials. The single feature change trials were identical to those in the block conditions. For the multi-feature change trials, all 3 features (syllable, pitch, and location) changed simultaneously. In order to balance overall performance with the single-feature change trials, the magnitude of the perceptual changes in the multiple-feature change trials was decreased. Specifically, the pitch of a multi-feature ‘different’ trial increased by 5 Hz (vs. 11 Hz in single-feature trials), the presentation location of the trial changed by ±50 μs (vs. ±100 μs in single-feature trials), and the syllable of the sound changed by +/− 4 steps (vs. +/− 8 steps in single-feature trials) on the ‘beet/bait’ continuum. As in the blocked conditions, subjects made a same/different confidence judgments for each trial, but in addition they also made a 3-alternative forced-choice ‘identification’ judgment (i.e., ‘what changed?’) in which they pressed ‘B’ if they believed a ‘beet/bait’ syllable change had occurred, ‘P’ if they believed a pitch change had occurred, or ‘L’ if they believed a location change had occurred. Subjects were instructed to make an identification judgment for each trial even if they were unsure.

### 2.2. Results

#### 2.2.1. Change detection ROCs

Change detection performance was examined by plotting receiver operating characteristics, and overall accuracy was measured as the Area Under the Curve (AUC). A paired *t*-test indicated that accuracy was not significantly different in the blocked and mixed list conditions t (145) = 1.096, *p* = .27) and so the results were collapsed across this factor for the main ROC analysis. The distribution of change detection confidence responses and change-identification responses in each condition are reported in Supplementary Tables 1 and 2.

**Fig. 3.**
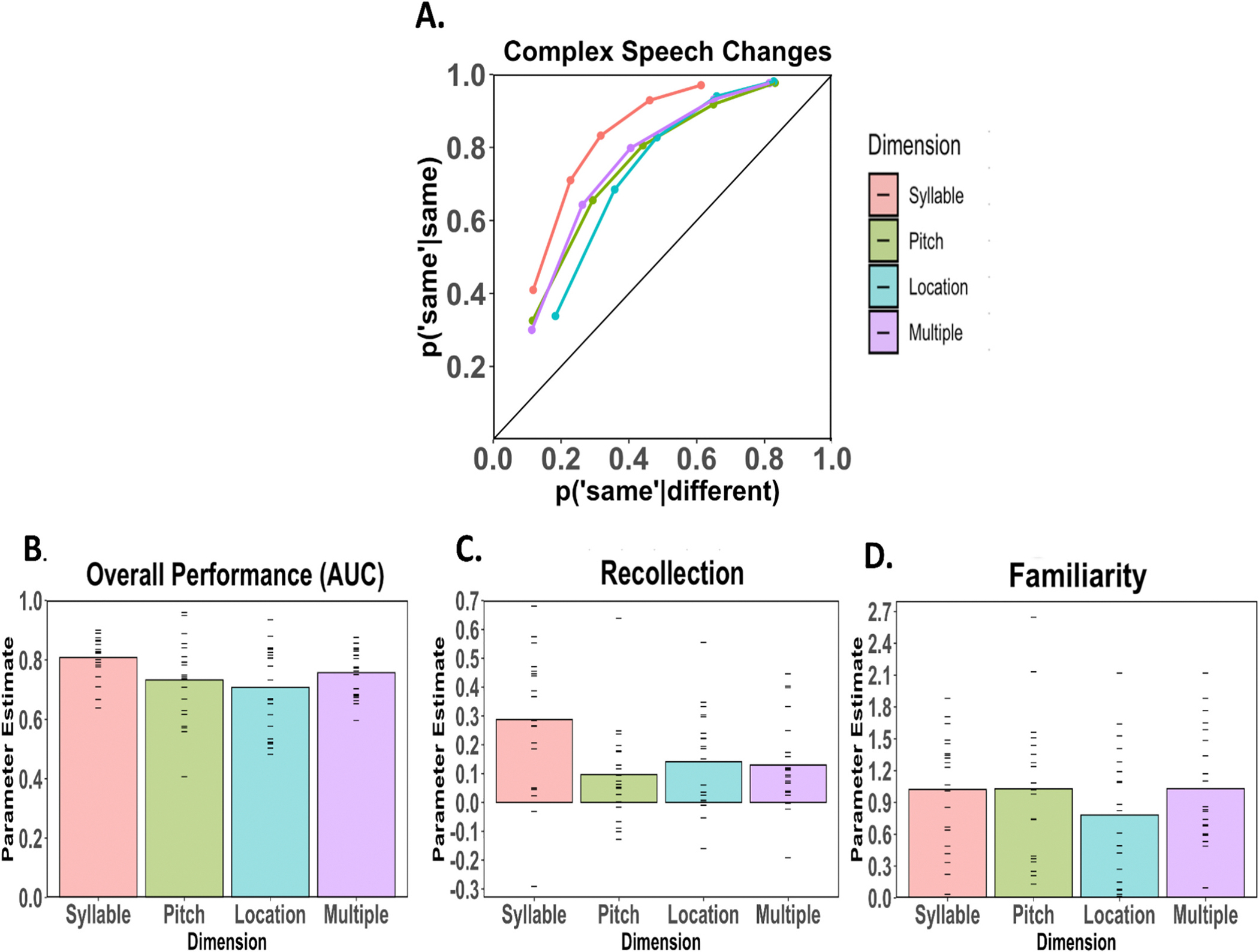
A-D. Change detection performance for speech sounds observed in Experiment 1. A) Average ROCs for the syllable-change, pitch-change, location-change and multiple-feature-change conditions. B) Average estimates of overall discriminability measured as area under the curve (AUC), with individual subject scores plotted as dashes. C) Estimates of recollection. D) Estimates of familiarity. Change detection performance was highest for the syllable-change condition and this was related to a higher likelihood of recollection in the syllable-change condition compared to the other types of changes. Familiarity was relatively constant across all of the change types.

The average ROCs for the syllable-change, pitch-change, location-change and multiple-feature-change conditions are plotted in Fig. 3. An examination of the figure shows that in each condition the ROCs were consistent with prior studies of visual working memory in the sense that the functions were curvilinear and asymmetrical (e.g., Aly & Yonelinas, 2012; Goodrich et al., 2019; Goodrich & Yonelinas, 2016). To quantify the degree of asymmetry we plotted the average ROCs in z-space and examined z-ROC slopes. The z-slopes were 1.44, 1.13, 1.39 and 1.19 for syllable, pitch, location and multiple-feature change conditions, respectively, indicating that the ROCs in each condition were asymmetrical (a symmetrical ROC has a z-slope of 1.0). The observed ROC asymmetry indicates that a single parameter model such as the equal variance signal detection model which predicts a slope of 1.0 is not sufficient to account for observed performance.

A further examination of the ROCs in Fig. 3 indicates that overall performance varied across conditions with better performance in the syllable-change condition than the other change conditions. Overall, memory performance as measured by the AUC is illustrated in Fig. 3B, and an analysis of variance revealed a significant difference in overall performance across the 4 change conditions (F_3,80_, = 3.05, *p* < .05). Follow-up pairwise comparisons with Tukey’s HSD revealed that overall charge-detection accuracy was higher in the syllable- than the location-change condition (*p* = .024), whereas there were no other significant pairwise differences between the other conditions (all other *p* > .1).

#### 2.2.2. Recollection and familiarity

To quantify the ROCs further we fit the dual process signal detection model to estimate recollection and familiarity (see Fig. 3C and 3D). It is common to constrain the memory parameters to be greater than 0, but here we allowed individual subject parameters to take on negative values, which led to negative recollection estimates in some subjects (see Fig. 3C). The reason we did this was to be consistent with the analyses conducted in experiments 2 and 3, which tested if stimulus-limited conditions could reduce estimates of recollection to 0, thus requiring the constraint to be relaxed. Critically, however, when we repeated the analysis with parameter estimates constrained to be non-negative, this did not change the current pattern of results.

An examination of model parameters indicated that there was a significant effect of change type on estimates of recollection (F(3, 80) = [4.42], *p* = .006; Fig. 3C). Follow-up pairwise comparisons revealed that the estimate of recollection in the syllable-change condition was significantly greater than in the pitch-change condition (p = .006), the multiple-feature-change condition (*p* = .03), and marginally greater than in the location-change condition (*p* = .06). In contrast to recollection, estimates of familiarity did not differ across conditions (F(3, 80) = [0.84], *p* = .47; Fig. 3D), and none of the pairwise comparisons across conditions were significant (all *p* > .2). To ensure that the results were not a consequence of allowing subject parameter values to be negative, we then constrained the parameters to be non-negative. As in the initial analysis, there was a significant effect of change type on recollection (*p* = .001) and no effect of change type on familiarity (p = .47). Overall, the results suggest that subjects were more accurate at detecting syllable changes than the other types of changes because of an increased likelihood of recollecting those types of changes, whereas familiarity-based discriminations were similar across the different change types.

#### 2.2.3. Change-identification accuracy

In the mixed list conditions, subjects made 3-alternative forced-choice responses to indicate which feature had changed, which allowed us to examine the subjects’ change-identification accuracy. Overall change-identification accuracy was significantly above chance (i.e., M = 0.55 > 0.33, t(40) = 7.432, *p* < .001), indicating that when making change detection judgments subjects were often able to correctly identify which feature had changed.

**Fig. 4.**
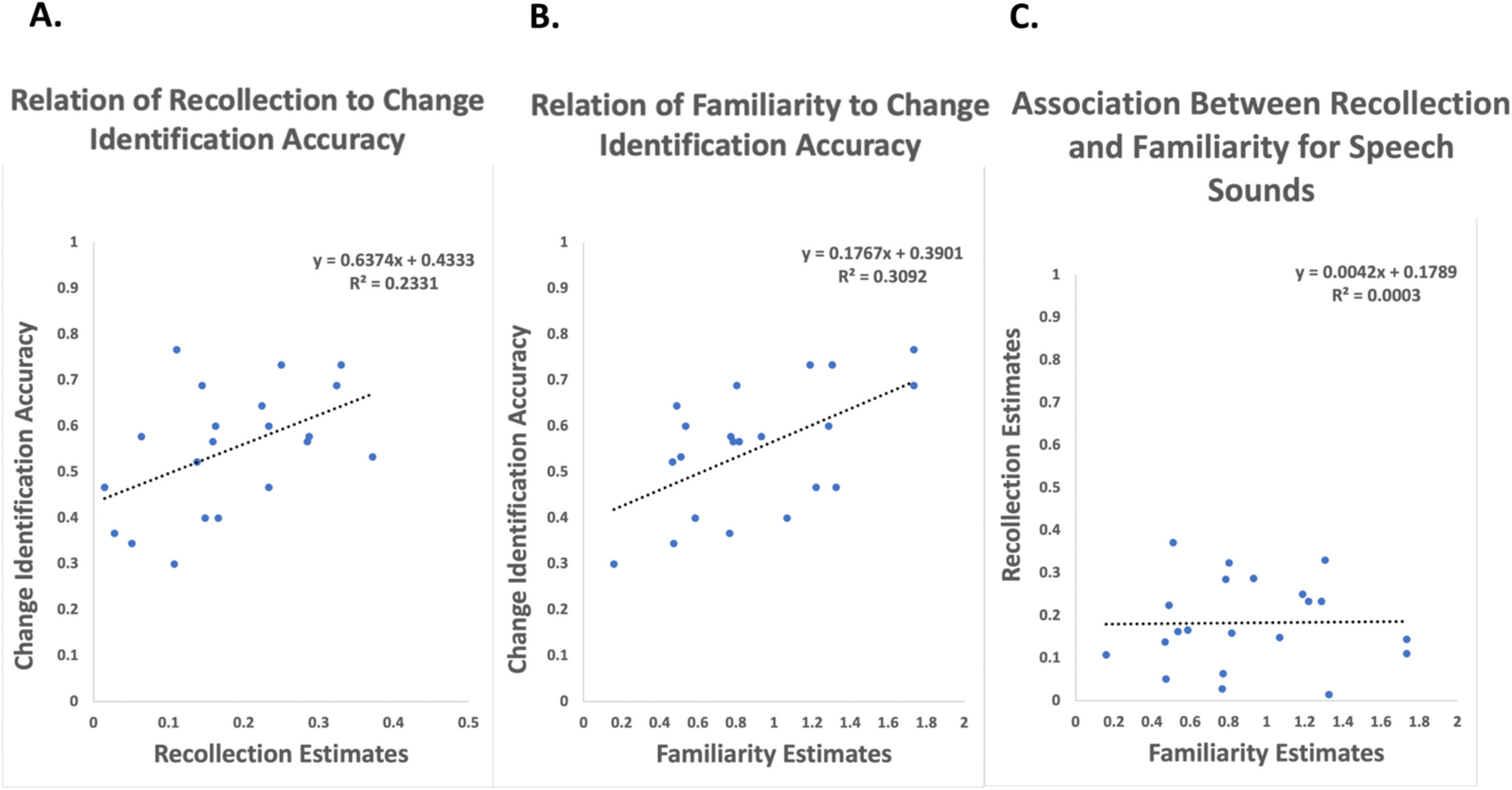
Change-identification accuracy and its relationship to the processes underlying change detection. A) Change-identification accuracy was positively related to estimates of recollection in change detection. B) Change-identification accuracy was also positively related to estimates of familiarity in change detection. C) Estimates of recollection and familiarity were not directly related to each other, suggesting that recollection and familiarity may play independent roles in change-identification.

To determine how change-identification accuracy was related to change detection we examined how subjects’ change-identification accuracy was related to their estimates of recollection and familiarity. Fig. 4 shows that higher levels of change-identification accuracy were related to higher estimates of recollection (Fig. 4A; t(19) = 2.403, *p* = .027), and to higher levels of familiarity (Fig. 4B; t(19) = 2.916, *p* = .008). Notably, recollection and familiarity estimates were not significantly correlated with one another (Fig. 4C; t(19) = 0.076, *p* = .94), suggesting that change-identification accuracy is independently related to recollection and familiarity. To further test this we used AIC model selection with estimates of recollection and familiarity as predictors of overall change-identification accuracy. The model with recollection and familiarity accounted for 90 % of the cumulative model weight, and reduced the AIC considerably (Burnham & Anderson, 2004) when compared to a model that included only familiarity (accounting for 0.07 % of the model weight, delta-AIC = +5.14), and when compared to a model that included only recollection (0.02 % of the model weight, delta-AIC = +7.33). This suggests that both recollection and familiarity contributed unique variance to change-identification. Thus, the results show that both recollection and familiarity-based change detection responses provide information not only about the occurrence of change, but about the specific features of the stimulus that changed.

In sum, the results of Experiment 1 indicated that the observed confidence ROCs were consistent with what has been observed in visual working memory (Aly & Yonelinas, 2012; Goodrich et al., 2019; Goodrich & Yonelinas, 2016). Namely, the ROCs in each condition were curvilinear as expected if a signal detection based familiarity process contributed to performance, and they were asymmetrical with a steep slope as expected if subjects were often able to recollect when a change had been made. In addition, the results indicated that subjects were better able to detect syllable changes compared to pitch or location changes, and that this was due to a selective increase in the proportion of recollected syllable changes, rather than reflecting an increase in familiarity. Thus, recollection is functionally dissociable from familiarity in auditory working memory. Furthermore, estimates of both recollection and familiarity were positively correlated with change-identification accuracy, but not one another, suggesting they each accounted for independent variance in the ability to identify which specific feature had changed.

**Fig. 5.**
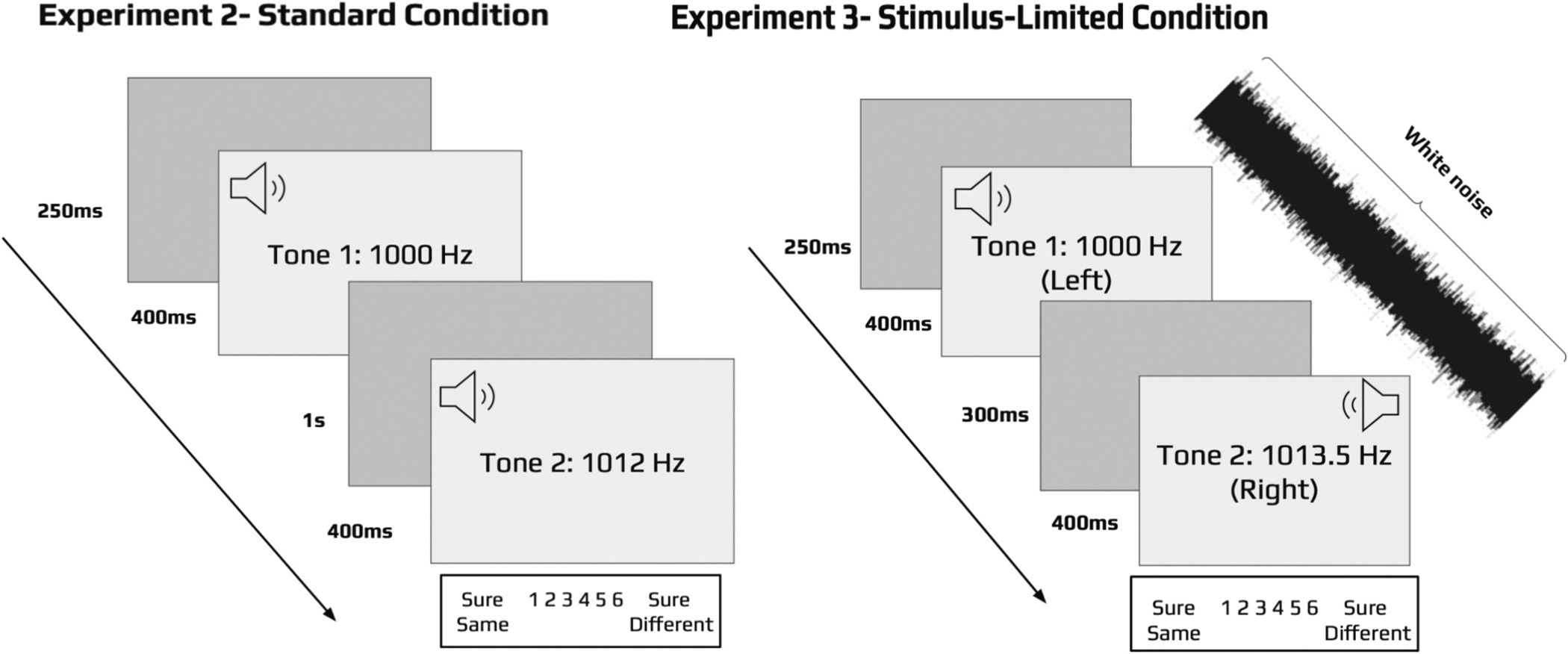
Illustration of the test procedures for Experiment 2 and 3. Subjects heard a pure tone followed by the same or changed tone and then they indicated if the tone was the same or different using a 6-point confidence scale. Experiment 3 was identical to Experiment 2 except that noise was introduced during each trial, the tones were presented to different locations (left then right ear) rather than centrally, and the study-test delay was reduced from 1 s to 300 ms.

Although Experiment 1 suggested that recollection and familiarity are required to account for working memory for speech-based stimuli, whether the same is true for simpler auditory materials like tones is unknown. In order to assess this, Experiment 2 examined change detection for tones in which the tones would either remain the same or change slightly in frequency. Otherwise, the test conditions were the same as those used in the blocked conditions in Experiment 1 (see Fig. 5). Experiment 3 was included to assess whether the processes underlying auditory working memory behaved in a manner similar to what has been observed in visual working memory. That is, in visual working memory, both recollection and familiarity contribute to change detection, however, recollection has been found to be eliminated under ‘stimulus-limited’ conditions. More specifically, Aly and Yonelinas (2012) found that when visual stimuli were made ‘just noticeable’ by using a visual mask and by rapidly presenting stimuli to different locations, the resulting ROCs were symmetrical and could be accounted for by a single familiarity process. The results were interpreted as indicating that stimulus-limited conditions were sufficient to support familiarity-based discriminations but were not sufficient to support conscious perception of the individual stimulus features that might change. If recollection and familiarity support similar functional roles in auditory memory as they do in visual memory, we expect that the ROCs in standard test conditions for auditory tones would be curved and asymmetrical (Experiment 2), whereas under stimulus-limited conditions the ROCs would become curved and symmetrical (Experiment 3). Experiment 3 was identical to Experiment 2 except that the tones were presented in rapid succession (i.e., interstimulus delay was reduced from 1000 ms to 300 ms), to different locations (i.e., the first and second stimuli in each trial were presented to the left and right ear respectively) and were masked with auditory noise (see Fig. 5). The only other difference between Experiments 2 and 3 was that the change magnitude was increased from 12 Hz to 13.5 Hz to ensure that overall difficulty was matched across experiments.

## 3. Experiments 2 and 3: working memory for tones

### 3.1. Methods

#### 3.1.1. Participants, materials, and procedure for experiment 2

The materials and methods were identical to those used in the blocked conditions in Experiment 1 with the following exceptions. Rather than testing perception for speech sounds, participants were tested on change detection for pure tones (e.g., 1000 Hz / 1012 Hz). During a ‘no change’ trial both stimuli were presented at the same frequency, whereas during a ‘change’ trial, the tone would increase or decrease by 12 Hz (i.e., 1012 Hz / 1000 Hz, 988 Hz / 1000 Hz, 1000 Hz / 988 Hz, or 1000 Hz / 1012 Hz). Twenty-four participants were recruited for the experiment, but 2 of the subjects were excluded from the analysis for failing to use a sufficient range of confidence responses, resulting in a final sample of 22 participants (15 female).

#### 3.1.2. Participants, materials and procedure for experiment 3

Twenty-five participants completed the experiment, but 5 subjects were excluded for failing to use a sufficient range of confidence responses, resulting in a final sample of 20 (10 female). The materials and procedures were identical to Experiment 2 with the following exceptions. In order to examine performance under stimulus-limited conditions (Aly & Yonelinas, 2012), the delay between the first and second tones in each trial was decreased from 1000 ms to 300 ms, each trial was presented along with a simultaneous uniform white noise mask, and the first tone was presented to the left ear followed by the second tone presented to the right ear. Finally, to equate the overall task difficulty to that of Experiment 2, the change magnitude was increased from 12 Hz to 13.5 Hz.

### 3.2. Results

#### 3.2.1. Change detection ROCs

**Fig. 6.**
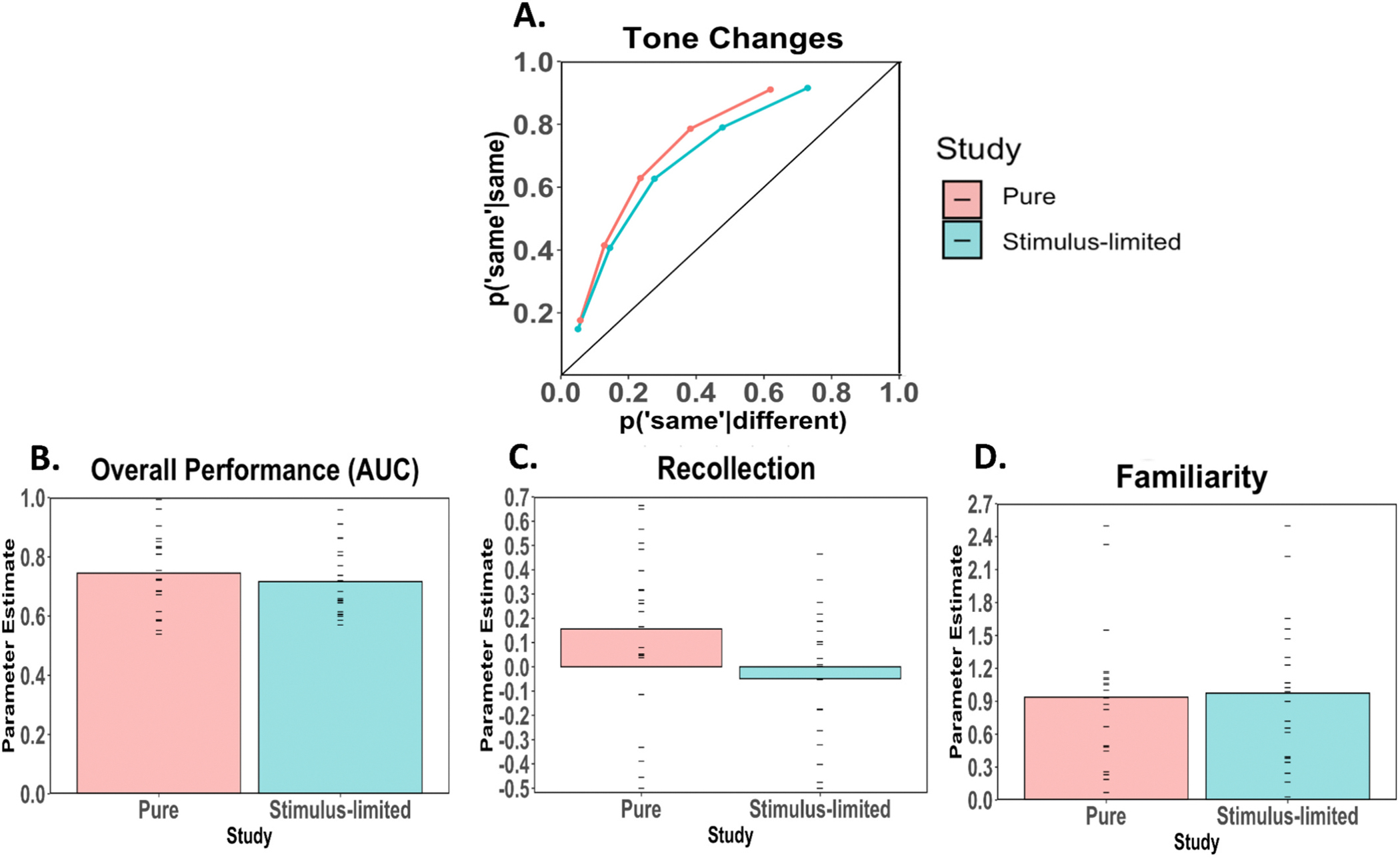
A-D. Change detection performance for tones observed in Experiments 2 and 3. A) Average change detection ROCs for pure tones under standard test conditions (Experiment 2) and stimulus-limited conditions (Experiment 3). Under standard test conditions the average ROC was curved and asymmetrical, whereas under stimulus-limited conditions the ROC was curved and symmetrical. B) Overall performance (i.e., area under the curve) for standard and data-limited conditions. C) Estimates of recollection. D) Estimates of familiarity. Change detection performance was similar across standard and stimulus-limited conditions, however, recollection was reduced to zero under stimulus-limited conditions whereas familiarity was unaffected.

The average ROCs for the pitch-change judgments of tones under standard test conditions (Experiment 2) and under stimulus-limited test conditions (Experiment 3) are plotted in Fig. 6.

The average ROC in the standard pure tone condition was curvilinear and asymmetrical, in line with the ROCs observed for the speech sounds in Experiment 1. Overall discriminability was comparable to that observed for speech sounds (i.e., in Experiment 2 the AUC was = 0.75 whereas in Experiment 1 the average AUC values varied between 0.71 and 0.81), and the degree of asymmetry was also comparable to that observed for speech sounds (i.e., in Experiment 2 average z-slope was 1.20 whereas in Experiment 1 the slopes were between 1.13 and 1.44).

In contrast, the average ROC in the stimulus-limited condition was symmetrical (i.e., the z-slope was 1.0), rather than asymmetrical as observed in the standard condition (i.e., the z-slope was 1.20). The AUC was slightly but not significantly lower in the stimulus-limited condition than in the standard condition (0.75 and 0.72; t(40) = −0.746, *p* = .50; Fig. 6B). The results indicate that tone detection and speech detection led to comparable ROCs. In addition, the ROC asymmetry associated with recollection was effectively eliminated for under stimulus-limited conditions.

#### 3.2.2. Recollection and familiarity

Estimates of recollection and familiarity for tone detention under standard and stimulus-limited conditions are presented in Fig. 6C and D. Recollection was significantly lower in the stimulus-limited condition compared with the standard condition (t(40) = −2.063, *p* = .02). In addition, recollection was significantly above zero in the standard condition (t(21) = 2.1201, p = .02), but was not different from zero in the stimulus-limited condition (t(19) = −0.744. In contrast to recollection, there was no significant difference in familiarity between the standard and stimulus-limited conditions (t(40) = 0.183, *p* = .85).

Experiment 2 indicated that working memory for pure tones produced ROCs that were similar to those observed for speech sounds (Experiment 1) in the sense that the ROCs were curvilinear as expected if familiarity contributed to performance and asymmetrical as expected if some auditory changes were recollected. In addition, as predicted on the basis of prior visual working memory results (Aly & Yonelinas, 2012), under stimulus-limited conditions (i.e., noise-masked, brief lateralized presentations in Experiment 3) the ROCs become symmetrical as expected if performance relied on a single familiarity process, and parameter estimates indicated that stimulus-limited compared to standard test conditions did not impact familiarity but eliminated the contribution of recollection.

## 4. Discussion

The current study examined the role of recollection and familiarity in auditory working memory. Experiment 1 examined working memory for speech-based sounds under conditions in which subjects detected changes in either the syllable, pitch, location or multiple features of the speech sounds. In each condition, the resulting ROCs were curvilinear and asymmetrical as expected if both recollection and familiarity contributed to performance. In addition, fitting the ROCs to the dual process signal detection model verified that both recollection and familiarity made contributions to performance for each change type. Moreover, the ROC analysis revealed that subjects more accurately detected syllable changes than the other types of auditory changes, and that this advantage was related to a selective increase in estimates of recollection, and not familiarity. Finally, in Experiment 1, estimates of both recollection and familiarity across subjects were positively correlated to change-identification accuracy, but were not correlated with one another, suggesting that both types of change detection responses were accounting for independent variance in change-identification performance. Experiment 2 examined change detection for pure tones and found curved asymmetrical ROCs that were similar to those observed with the speech sounds in Experiment 1. Experiment 3 also examined change detection for pure tones but under stimulus-limited conditions (i.e., brief delay, lateralized presentations with noise masking), and revealed curved ROCs, but in this case the ROC was symmetrical, as expected if performance relied solely on familiarity. This dissociation was predicted based on similar results in visual working memory.

The present results build on prior studies of visual and auditory working memory but extends that work in several important ways. For example, although a growing body of working memory research has indicated that both recollection and familiarity contribute to change detection performance, those studies have focused almost exclusively on visual materials (e.g., Aly & Yonelinas, 2012; Goodrich et al., 2019; Goodrich & Yonelinas, 2016, 2020; Ramey et al., 2022; Yonelinas et al., 2024). One previous ROC study of auditory working memory found that ROCs related to changes in auditory scenes were curved and asymmetrical, which is consistent with the role of recollection and familiarity (McAnally et al., 2010), but that study examined only complex auditory scenes and it was not designed to determine if recollection and familiarity were functionally distinct. If recollection and familiarity reflect distinct auditory memory processes as they do in visual working memory, then it should be possible to find experimental manipulations that influence one of the processes without influencing the other. Alternatively, they may simply reflect two aspects of the same underlying memory signal that always increase or decrease together. The current study showed that recollection and familiarity contributed to auditory working memory for a variety of auditory materials ranging from syllable changes in speech sounds to frequency changes in pure tones. Most importantly, the results indicated that the two processes can be functionally dissociated, in the sense that better performance in detecting syllable changes compared to pitch or location changes was related to a selective increase in recollection, and that stimulus-limited conditions selectively eliminated recollection while leaving familiarity intact, as has been observed in studies of visual working memory. Additionally, an examination of change-identification performance in Experiment 1 showed that both recollection and familiarity were independently related to the ability to correctly identify changed features, consistent with the notion that the two processes serve independent, but complementary, functions in working memory. Thus, the emerging picture is that recollection and familiarity reflect functionally distinct memory processes that play critical roles in working memory across many different types of stimuli and across both visual and auditory domains.

In the current experiments we used the ‘dual process’ signal detection model to derive estimates of recollection and familiarity. An alternative model is the ‘equal variance’ model which includes a single familiarity parameter (d’) to estimate memory strength. This model, however, predicts symmetrical ROCs and so it is inconsistent with the observed ROCs. However, another alternative is the ‘unequal variance’ model. This model can produce ROCs that are quite similar to the dual process model (footnote 1), but in the current study it leads to conclusions that are broadly consistent with those based on the ‘dual process’ model. That is, the unequal variance model is based on the same familiarity process as the dual process model (i.e., an equal variance signal detection process), but rather than assuming that there is a separate recollection process that contributes to high confidence change detections, it assumes there is a process that increases the variance of the changed item familiarity distribution and this is what produces the ROC asymmetry. One interpretation of this second component is that it reflects a recollection-like process that impacts the new items more so than the old items and so it leads the new item distribution to have greater variance (i.e., the ROC asymmetry). As such, the conclusions one would draw if one adopted this approach are similar to that of the dual process model. For example, the observation that the recollection parameter decreased while the familiarity parameter was unaffected, would correspond to the observation that variance parameter was decreased while the familiarity parameter was unaffected.

A second interpretation of the unequal variance signal detection model is that the extra variance associated with changed items compared to the same items, does not reflect recollection per se but rather some other memory-related process that selectively increases the variance of the familiarity strength distribution for changed items, relative to the same items. For example, because the change trials reflected a mixture of trials in which there was an ‘increase’ along the stimulus dimension and others where there was a ‘decrease’ along the stimulus dimension, these two types of changes may not have been psychologically equivalent, and this may have increased the observed variance in the changed trials. For example, a decrease from 1000 Hz to 988 Hz may not be psychologically equivalent to an increase from 1000 Hz to 1012 Hz. In this way, the variance in familiarity for the changed trials could be greater than variance in familiarity for the same trials where the tone remained constant. That is, the total variance for changed trials is equal to the “within-stimulus” variance for a single change stimulus (here, a tone at some frequency) *plus* the “between-stimuli” variance owing differences in the mean perceptual evidence of a change (i.e., d’) produced each of the change stimuli relative to its comparator. However, such an account runs into a few problems. First, the differences in variability expected on the basis of the stimuli that were used in the current study were likely insufficient to have produced the observed differences in memory variances. Expanding on the pure tone example, the just-noticeable difference (i.e., difference limen) of frequency discrimination within the 988 Hz to 1012 Hz range can be assumed to be a constant proportion of the equivalent rectangular bandwidth (ERB; Sek & Moore, 1995). A calculation of ERBs for our 1000 Hz to 988 Hz change stimulus, and our 1000 Hz to 1012 Hz change stimulus, produces ERB values of 0.0912 and 0.0903, respectively. Assuming proportionality of d’ with change magnitude expressed in ERBs, this suggests the “between-stimuli” variance component is negligible. The effect of this variance component on ROC shape can be formally estimated by treating the memory strength distribution on change trials as a mixture of equal variance Gaussian distributions. For our tone experiment with two change stimuli, the cumulative distribution function for a mixture of two equal variance Gaussians is:

$${CDF}_{\text{Change}}=0.5+0.25^{*}ERF\left(\frac{\mu_{1}\;-\;c}{2}\right)+0.25^{*}ERF\left(\frac{\mu_{2}\;-\;c}{2}\right)$$

where $\mu_{1}$ is the mean change strength produced by the 1000 Hz to 988 Hz stimulus, $\mu_{2}$ is the mean change strength produced by the 1000 Hz to 1012 Hz stimulus, c is the decision criterion, and ERF is the error function. If we further treat the distribution for no-change trials as univariate Gaussian with mean $\mu_{0}$, constraining the sum of $\mu_{0},\mu_{1}$, and $\mu_{2}$ to be zero, we can generate ROCs for different assumed levels of “between-stimuli” variance by changing the ratio of d’ for the 1000 Hz to 988 Hz stimulus $\left(\mu_{1}-\mu_{0}\right)$ and the 1000 Hz to 1012 Hz stimulus $\left(\mu_{2}-\mu_{0}\right)$. With no “between-stimuli” variance, setting both d’ equal to 1.35 produces a perfectly symmetric, curvilinear ROC with z-slope 1 and AUC 0.75 (i.e., the approximate mean AUC for our empirical ROCs in the tone experiment). If we set the ratio of d’ to 2, such that d’ for the 1000 Hz to 988 Hz stimulus is 0.933 and d’ for the 1000 Hz to 1012 Hz stimulus is 1.867, this produces a slightly asymmetric ROC with AUC maintained at 0.75. However, the z-slope is only 0.95, corresponding to a recollection estimate of ~0.06 if we apply the dual process model. If we set the ratio of d’ to 10 (d’ for the change stimuli of 0.318 and 3.18, respectively), this maintains AUC of 0.75 and produces an ROC with a z-slope of 0.74, corresponding to a recollection estimate of 0.35 approaching the mean estimate for the empirical data. However, this assumed difference in d’ is clearly incompatible with the empirical data, suggesting that “between-stimuli” variance alone cannot be responsible for our findings. The case for a substantial contribution of “between-stimuli” variance is stronger for the speech experiment, particularly for the syllable change condition where there were multiple change stimuli with plausibly large differences in mean change strength (e.g., crossing versus not crossing the phoneme category boundary). However, the speech experiment remains compatible with the dual process model (e.g., with recollection more likely to occur on cross-category versus within category changes) and, given the pure tone example above, increased contributions of “between-stimuli” variance would not undermine the fundamental claim for a contribution of recollection to auditory working memory.

In addition, the account fails to explain why the difference in variance was eliminated in Experiment 2 under stimulus limited conditions - an effect that was predicted by the recollection account. One could, however, propose a different post hoc explanation of this result. For example, perhaps the stimulus limited condition made the task more perceptually demanding and so selectively led to an increase in variance of the same trials that perfectly offset the increase in variance for the changed trials that should have arisen because of differences in the change item strength produced by the mixture of increased and decreased tones. Although it is difficult to rule out this type of post hoc account, we don’t find it particularly compelling or useful. Nevertheless, we do think that additional studies that directly test competing predictions of the dual process and unequal variance models would be informative. For example, although the two models can produce similar shaped ROCs, under conditions in which performance is more heavily reliant on recollection the dual process model predicts that the ROCs should become flatter and exhibit a noticeable U-shape when plotted in z-space. In contrast, the unequal variance accounts predict that the z-ROCs should be linear (otherwise the underlying Gaussian assumption underlying the model must be modified). In studies of visual working memory, the observed z-ROCs are often U-shaped, thus supporting the dual process model prediction (Aly & Yonelinas, 2012; Yonelinas, 2023). In addition, in auditory working (McAnally et al., 2010) the ROCs were found to fit significantly better by the dual process than the unequal variance signal detection model. However, whether the same will hold with simpler auditory materials like those examined in the current study is not yet known.

Footnote 1: Direct comparisons of these two models indicated that they both provided equally good accounts of the observed ROCs in the current studies (i.e. in Experiment 1 the average SSEs for the dual process and unequal variance signal detection models were 0.0028 and 0.0021, respectively (*p* > .05), in Experiment 2 the SSEs were 0.003 and 0.002 (p > .05), and in Experiment 3 the respective SSEs were 0.002 and 0.002 (p > .05)).

How do the current results inform our theories of working memory? At the broadest level, the results suggest that working memory across both visual and auditory domains can be supported by two complementary memory processes: recollection and familiarity. Although a number of previous theories of working memory have proposed material-specific distinctions within working memory such as distinctions between visual and phonological working memory (Baddeley, 2000; Baddeley & Hitch, 1974), verbal and tonal working memory (Deutsch, 1970; Schulze et al., 2018), and visual and spatial working memory (Klauer & Zhao, 2004), these theories have not distinguished between recollection and familiarity. In fact, a number of theories have assumed that working memory reflects a recollection-like maintenance process, whereas others have assumed working memory reflects a familiarity-like memory matching process. For example, a number of cognitive psychologists characterize working memory as an active subjectively accessible rehearsal process that is capacity limited such that only a limited number of items can be remembered (Atkinson & Shiffrin, 1968; Waugh & Norman, 1965). This work was further extended to propose both visual and phonological rehearsal buffers (Baddeley & Hitch, 1974). In contrast, with the development of signal detection based theories of decision making (Swets et al., 1961), other memory theorists (e.g., Murdock Jr., 1965) focused more on memory strength based approaches to working memory whereby studied items were treated as being more familiar than non-studied items. These approaches assumed that there was no fundamental difference between remembered and non-remembered items, only differences in memory strength. There are still active debates about which of these approaches is correct (Bays & Husain, 2008; Schurgin et al., 2020; Wilken & Ma, 2004) The current results suggest that both approaches may be partially correct, and that subjects are able to utilize both types of memory processes to support working memory responses. Moreover, the current results suggest that this is true across a variety of different types of stimuli, and across both visual and auditory materials.

The results should not be seen as arguing that there are no material-specific forms of working memory. However, we argue that within each of these types of working memory there is a more general distinction between recollection and familiarity-based responses. Moreover, although we have focused on auditory (and previously on visual) working memory we speculate that the same two processes may also be involved in other domains, such as tactile, olfactory, or motor working memory tasks. This will of course need to be assessed in future studies.

The proposal that working memory relies on recollection and familiarity shares some similarities with two other multiple-component models of working memory. For example, Cowan has suggested that working memory reflects the contents of a limited capacity focus of attention, as well as activation of long-term semantic memory representations (Cowan et al., 2020). Whereas, Oberauer has argued that working memory reflects direct access to a context frame that temporarily links studied items together, as well as activation of long-term semantic memory representations (Oberauer, 2013). Although neither of these models make claims about memory confidence or subjective experience per se, it is reasonable to assume that sustained attention may be critical in supporting conscious recollection of qualitative information, and that it may carry with it some forms of contextual information linking the maintained items together. In addition, activation of semantic representations may give rise to a familiarity signal that is consistent with a signal detection-like process. However, we note that the current approach differs in the sense that familiarity is not assumed to be limited to activation of existing representations, but rather suggests that it supports a global matching signal and as such should support novel associative learning. Future work contrasting these approaches will be informative.

The present results also raise a number of additional questions that will need to be addressed in future studies. For example, the finding that subjects in the current study were better able to detect syllable changes than pitch or location changes, and that this reflected a selective increase in recollection, suggests that syllable changes preferentially led to recollection. One possible account of this is that syllable changes are more complex than pitch or location changes, and so change complexity may be more critical for recollection than familiarity. Alternatively, syllable changes may rely on more categorical or speech-based representations (i.e., lexical or phonological) than do pitch or location changes and so it could be the categorical or speech-based nature of the changes that made them more amenable to recollection. In addition, the finding that presenting tones under stimulus limited conditions (i.e., short delay, lateralized and noise masked) effectively eliminated recollection, but did not impact familiarity, indicates that recollection is less likely to occur under these test conditions. Future studies will be needed to further examine exactly which of the manipulated aspect(s) are necessary for recollection to arise.

Do recollection and familiarity also play a role in other change detection paradigms? The current results suggest that for speech-based sounds and simple tones, change detection decisions reflect the combined effects of recollection and familiarity processes. These effects seem to hold whether subjects are required to detect simple sensory discriminations like changes in pitch or in location, or more complex changes such as changes in syllable information. Moreover, similar results were obtained when subjects were required to attend to a single feature change and when they had to attend to multiple auditory features. Whether these results generalize to other more complex auditory change detection tasks should be explored in future studies, but based on a consideration of some related work, we suspect that these results will be general. For example, prior studies of ‘visual’ working memory have indicated that both recollection and familiarity contribute to change detection across a wide range of conditions, ranging from detecting color changes in Gabor patches (Goodrich et al., 2019) to detecting changes in complex visual scenes (Aly & Yonelinas, 2012). In addition, as mentioned above, one earlier ROC study found that in a change detection task in which subjects were required to maintain multiple naturalistic sound objects in a complex auditory scene, both recollection and familiarity were also found to support performance (McAnally et al., 2010). Importantly, however, future studies which use more complex stimuli are needed to firmly answer the question of generalization.

Finally, future work should be aimed at elucidating the extent to which recollection and familiarity in auditory working memory are related to subjective reports of these processes, as they are in visual working memory studies (Aly & Yonelinas, 2012). Additionally, work showing a reverse dissociation (i.e., eliminating familiarity without compromising recollection) would provide strong evidence in support of our hypothesis that memory is supported by two independent, complementary processes. Moreover, the neural substrates associated with recollection and familiarity in auditory working memory should be explored. For example, how might the two processes be affected in healthy and pathological aging? Or by lesions to regions such as the medial temporal lobe or lateral-parietal cortex?

In light of the large body of research outlining the distinctions between visual and auditory working memory, the present study expands what we know about the processes that support working memory across various domains. Specifically, the present study shows that models of working memory performance must account for the independent contributions of both recollection and familiarity, regardless of sensory domain, and that both processes contribute to auditory working memory for a variety of stimulus types.

## Supplementary Material

Supp

Appendix A. Supplementary data

Supplementary data to this article can be found online at https://doi.org/10.1016/j.cognition.2024.105987.

## Data Availability

Data available upon request.
