# Supplementary material for: Recollection and familiarity support auditory working memory in a manner analogous to visual working memory
Source: Cognition. Author manuscript; Available in PMC 2025 Jul 18. (PMC12273585; doi:10.1016/j.cognition.2024.105987)
Supplement: Supp [file NIHMS2093867-supplement-Supp.docx]

| **Mixed** | **1** | **2** | **3** | **4** | **5** | **6** |
| --- | --- | --- | --- | --- | --- | --- |
| Same | 569 | 649 | 295 | 252 | 85 | 45 |
| Syllable | 47 | 65 | 64 | 117 | 96 | 236 |
| Pitch | 67 | 107 | 116 | 148 | 94 | 98 |
| Location | 129 | 136 | 76 | 125 | 89 | 75 |
| Multiple | 215 | 282 | 269 | 463 | 310 | 351 |

| **Blocked** | **1** | **2** | **3** | **4** | **5** | **6** |
| --- | --- | --- | --- | --- | --- | --- |
| Syllable-Same | 327 | 163 | 56 | 38 | 24 | 22 |
| Pitch-Same | 221 | 200 | 91 | 58 | 46 | 14 |
| Location-Same | 237 | 221 | 82 | 58 | 23 | 9 |
| Syllable-Different | 101 | 73 | 48 | 64 | 94 | 250 |
| Pitch-Different | 79 | 117 | 70 | 114 | 135 | 115 |
| Location-Different | 102 | 84 | 81 | 97 | 123 | 143 |

Supp. Table 1. Number of responses at each level of confidence (i.e., 1-6) and for each change type for the mixed and blocked conditions.

| **Change**  **Identification**  **Accuracy** | **1** | **2** | **3** | **4** | **5** | **6** |
| --- | --- | --- | --- | --- | --- | --- |
| Syllable | 0.23 | 0.41 | 0.17 | 0.43 | 0.74 | 0.85 |
| Pitch | 0.67 | 0.43 | 0.44 | 0.58 | 0.60 | 0.50 |
| Location | 0.50 | 0.25 | 0.39 | 0.54 | 0.79 | 0.88 |

Supp. Table 2. Change identification accuracy for each dimension at each level of confidence (1-6).
